# Spontaneous fracture and migration of catheter of a totally implantable venous access port via internal jugular vein - a case report

**DOI:** 10.1186/s13019-016-0450-y

**Published:** 2016-04-11

**Authors:** Seung Yeon Ko, Sun Cheol Park, Jeong Kye Hwang, Sang Dong Kim

**Affiliations:** Department of Surgery, College of Medicine, The Catholic University of Korea, Seoul, Korea; Department of Surgery, Incheon St. Mary’s Hospital, College of Medicine, The Catholic University of Korea, 56 Dongsu-ro, Bupyong-Gu, Incheon, 403-720 Korea

**Keywords:** Catheter fracture, Internal jugular vein, Totally implantable venous access port

## Abstract

**Background:**

The totally implantable venous access ports (TIVAPs) are indicated for patients undergoing chemotherapy, total parenteral nutrition and long-term antibiotic treatment. But, among their complications, the fracture and migration of the catheter of a TIVAP via internal jugular vein represents a very rare but potentially severe condition.

**Case presentation:**

A 50-year-old woman indentified with a spontaneous fracture and migration of catheter of a TIVAP via right internal jugular vein after adjuvant chemotherapy for ovary cancer. She had been not evaluated and not managed with the heparin lock flush solution during three months after adjuvant chemotherapy. And then, she complained right neck bulging during saline infusion via a TIVAP and a chest radiography showed the fractured and migrated catheter of a TIVAP in right atrium. So, we emergently removed the catheter fragment by a goose neck snare via right femoral vein. After then, there was no problem.

**Conclusions:**

If the fractured catheter of a TIVAP is detected, it is desirable to remove a fragment by an endovascular approach if it is possible.

## Background

A totally implantable venous access port (TIVAP) plays a crucial role in the treatment of patients in oncology [1]. But, among the late mechanical complications, lesions of the catheter wall represent a rare but potentially severe condition, whose natural history can vary from a partial rupture or catheter malfunction to a complete catheter fracture with embolization of the ruptured fragment [2]. Mostly, transection and embolization of a TIVAP, so called “Pinch-off” syndrome (POS), is a rare complication of a TIVAP via subclavian vein (SCV) and the incidence is reported to be 1.1–5.0 % (%) of patients with TIVAP via the SCV [3, 4]. But, a spontaneous fracture and migration of a TIVAP via internal jugular vein (IJV) is the more rare event. So, we report a case indentified with a spontaneous fracture and migration of catheter of a TIVAP via right IJV after adjuvant chemotherapy for ovary cancer and successfully retrieved by percutaneous endovascular approach.

## Case presentation

**Fig. 1:**
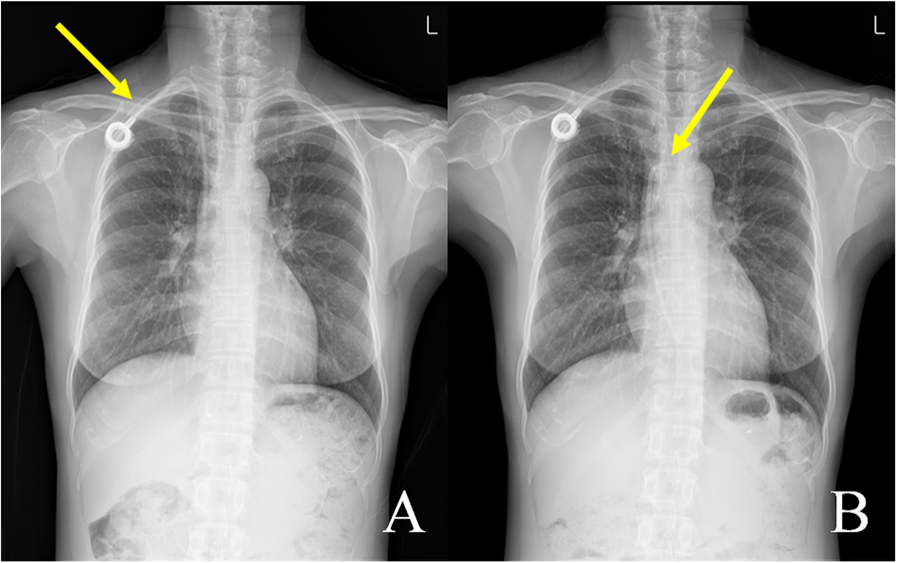
A totally implantable venous access port (TIVAP) on chest radiography. **a** Chest radiography shows a TIVAP locates at a appropriate position after TIVAP placement procedure. Arrow indicates a TIVAP. **b** Chest radiography shows fracture and migration of the catheter of a TIVAP. Arrow indicated the fractured catheter fragment moved into right atrium

**Fig. 2:**
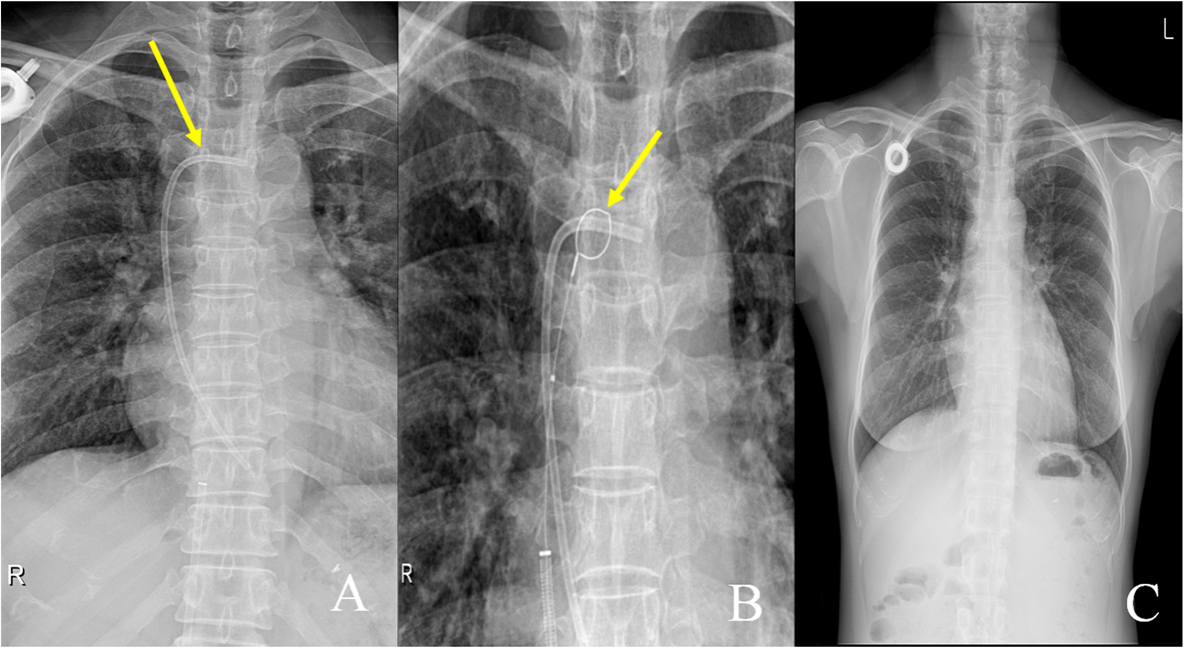
A procedure of removal of fractured catheter of a totally implantable venous access port (TIVAP). **a** Chest radiography identified the location of a fractured and moved part of a TIVAP. Arrow indicates the fractured catheter fragment. **b** A fractured and moved part of a TIVAP was caught by a goose neck snare with angiographic guidance. Arrow indicates a goose neck snare. **c** The fractured and moved catheter was completely removed

A 50-year-old woman was diagnosed with left ovary cancer and underwent an extended total hysterectomy with lymph node dissection, total omentectomy, and resection of splenic metastasis. On 1 week after a surgery, she was performed a TIVAP (DistricAth, 9 Fr, Districlass Medical SA, Chaponnay, France) placement via right IJV with a duplex ultrasonography for a chemotherapy. After then, a function of TIVAP was good, and a chest radiography showed the TIVAP located at the optimal position (Fig. 1a). So, she had been performed a chemotherapy via this TIVAP by a planned schedule. After the completion of a chemotherapy, she had been not followed-up for 3 months. During that period, TIVAP had been not evaluated and not managed with the heparin lock flush solution. During 3 months, she refused to be followed-up and didn’t visit our hospital. But, after 3 months, she visited an emergency room (ER) of our hospital with complaining an abdominal pain. In the ER, a doctor tried to infuse saline with a 10 mL syringe via TIVAP for a fluid therapy, but she complained a right neck bulging during saline infusion via TIVAP. So, we quickly checked a chest radiography. It showed the TIVAP had been fractured at the junction between the port and the catheter, and the fractured catheter was migrated into right atrium (Fig. 1b). There was no special etiology such as trauma. She obviously denied a history of trauma, and she had a little sports activity and was a housewife. And then, we emergently tried to remove the fractured catheter. We inserted a 7 Fr sheath into right femoral vein by a percutaneous puncture under local anesthesia. The fractured catheter fragment was subsequently caught and moved into the right femoral vein using a goose neck snare (Cook Inc, Bloomington, IN, USA) (Fig. 2a, b). And then, the fractured catheter fragment was removed successfully with venous sheath (Fig. 2c). Venous access site was compressed manually. After the confirmation of hemostasis on venous puncture site, the remnant port was removed surgically under local anesthesia. There was no complication during these procedures.

## Discussion

A catheter fracture with subsequent migration is a rare complication after the TIVAP implantation [5]. But, rupture of the catheter is potentially severe complication [2]. The causes of a catheter fracture of the TIVAP are unclear, but might include the following [6]. First, degradation and alteration in the meachnical properties of the catheter material, probably caused by the drugs administered. Second, flushing of the catheter should be performed gently using a 10 mL syringe to prevent catheter fracture because smaller syringes generate greater pressure, which increases the risk of rupture. Third, chronic stress against the catheter induced by the motion of the neck, clothing with a stiff collar or jewelry, and a safety belt or strap of the backpack, could affect catheter wall structure and cause the catheter fractures. Lin et al. [7] reported rates of port rupture of 2.17 %; all ports had been inserted via SCV, and the most common site of fracture was located at the junction between the injection port and the catheter. It can happen when a catheter is compressed vigorously and repeatedly between the clavicle and the first rib in the case of insertion via SCV [8]. In 1984, Aitken and Minton [9] first described the “pinch-off sign” (compression of the catheter crosses between the clavicle and the first rib) on chest radiography with a case of catheter fracture and embolization. But, all of them were inserted via SCV [8]. The incidence and magnitude of catheter ruptures are extremely variable, covering clinical situations which include asymptomatic ruptures, catheter malfunctions, pinch-off syndrome, full fracture with intravascular embolization of a catheter fragment [2, 10]. The pinch-off syndrome has been used to explain the fracture of catheters inserted through the SCV [4]. And, to prevent the occurrence of catheter fracture of TIVAP via SCV, many researchers recommended that the catheter of TIVAP was inserted through the right IJV [4, 11]. So, I think that anatomical location representing with the pinch-off syndrome is the most important reason of a difference between TIVAPs via IJV and TIVAPs via SCV. And, next reason is that ultrasound-guided TIVAP implantation via IJV has a higher success rate and fewer complications than unguided TIVAP implantation via SCV [12]. And, ultrasound-guided insertion also enables precise venopuncture and catheter positioning. So, we can decrease number of venopuncture and risk of vein injury, acute angulation of catheter, and catheter injury [12]. The incidence of catheter fracture by the pinch-off syndrome in TIVAPs via SCV is reported to be 1.1–5.0 % [3, 4]. Bucki et al. [13] reported that the catheter fracture rate of TIVAPs via right IJV was 0.7 % (2/309). Nagasawa et al. [6] reported that the catheter fracture rate of TIVAPs via right IJV was 1.8 % (1/106). Wu et al. [11] reported that the overall catheter fracture rate of TIVAPs was 3.92 % (59/1505) and 59 catheter fractured patients comprised of 26 patients (44.1 %) via right SCV, 22 patients (37.3 %) via right cephalic vein, 7 patients (11.9 %) via left cephalic vein, and 1 patient (1.7 %) via right IJV. And, the catheter fracture rate via right SCV was 13.4 % (26/194) and that via right IJV was 2.1 % (1/48) [11]. So, they reported that the implantation via the SCV route was a significant risk factor for catheter fracture of TIVAP (*P* = 0.0001) [11].

Features of catheter fracture and migration after TIVAP insertion via IJV were reported into the type of catheter (closed tip catheter), the site of the lesion (entry point into the vein wall) and the type of ultrasound approach (“out-of-plane” puncture of the IJV) [2]. Pressures developed by peristaltic pumps commonly used for chemotherapy may exceed 50 lb per square inch (psi), the pulse of the peristaltic pumps could exert a chronic mechanical stress, resulting in damage to the most vulnerable portion of the catheter wall [2, 7, 14]. In a closed tip catheter, it can be a more amplified situation. In this sense, the entry through the vein wall may represent the point of maximal catheter angulation, which generates maximal resistance to flow, as well as the site of maximal mechanical tension [2, 7, 14]. Furthermore, “out-of-plane” ultrasound-guided puncture of the IJV is invariably associated with a more vertical pathway and a narrower angle at the entry point into the vein wall [2]. All these factors could have played an important role in determining the lesions [2]. In our case, we used open tip catheter, and did “in-plane” ultrasound-guided puncture of the IJV. But, our patient has been not followed-up for 3 months after the completion of a chemotherapy. During that period, TIVAP had been not managed with the heparin lock flush solution. So, a thrombosis was formed in the catheter, and the pressure developed by a thrombosis could have a role in the fracture of a catheter.

An interesting issue is more than 50 % of fractured ports were clinically asymptomatic, and catheter lesions were accidental findings during scheduled port removal [2]. This confirms the fact that the natural history of the catheter damage cannot be adequately predicted, and the risk that these asymptomatic fractures might lead to more severe events cannot be excluded [2].

To prevent a catheter fracture of the TIVAP, we need a standardized venous port implantation and postoperative care in strict accordance with the operating specifications [15]. So, we have to insert the open-ended catheter by “in-plane” ultrasound-guided puncture of the IJV [2]. During implantation, an extreme change of direction should be avoided because this is where breakage can happen, and catheter damage and improper connections have to be also avoided [5, 15]. Venous ports require 100 unit/ml heparin flushes after each use or monthly during times of port inactivity [10]. Flushing of the catheter should be done gently with a 10 mL syringe [3, 6]. If a patient has painful swelling of the TIVAP insertion site and clavicular area soon after flushng, a chest radiograph must be taken to investigate the possibility of a fractured catheter [3]. Because of the risks of arrhythmia, septic complication, thromboembolism, and cardiac arrest, the fragment should be removed as quickly as possible [8]. Early detection and endovascular removal might provide the opportunity for prevention of further complications and surgical techniques such as thoracotomy for removal of the catheter [8].

## Conclusion

We need to do regularly follow-up with chest radiography and manage with the heparin lock flush solution for a TIVAP. And, if a TIVAP is unuseful after a scheduled treatment, we need to remove it. If the fractured catheter of a TIVAP is detected, it needs to do the prudent evaluation of the fractured fragment. After that, to prevent further complications, it is desirable to remove a fragment by an endovascular approach if it is possible.

## Consent

Written informed consent was obtained from the patient for publication of this Case report and any accompanying images. A copy of the written consent is available for review by the Editor-in-Chief of this journal.

## Abbreviations

IJV: internal jugular vein
POS: pinch-off syndrome
SCV: subclavian vein
TIVAP: totally implantable venous access port
